# µ-Conotoxins Modulating Sodium Currents in Pain Perception and Transmission: A Therapeutic Potential

**DOI:** 10.3390/md15100295

**Published:** 2017-09-22

**Authors:** Elisabetta Tosti, Raffaele Boni, Alessandra Gallo

**Affiliations:** 1 Department of Biology and Evolution of Marine Organisms, Stazione Zoologica Anton Dohrn, Villa Comunale, 80121 Naples, Italy; elisabetta.tosti@szn.it; 2 Department of Sciences, University of Basilicata, 75100 Potenza, Italy; raffaele.boni@unibas.it

**Keywords:** conotoxin, µ-conotoxin, ion current, sodium channel, pain transmission

## Abstract

The Conus genus includes around 500 species of marine mollusks with a peculiar production of venomous peptides known as conotoxins (CTX). Each species is able to produce up to 200 different biological active peptides. Common structure of CTX is the low number of amino acids stabilized by disulfide bridges and post-translational modifications that give rise to different isoforms. µ and µO-CTX are two isoforms that specifically target voltage-gated sodium channels. These, by inducing the entrance of sodium ions in the cell, modulate the neuronal excitability by depolarizing plasma membrane and propagating the action potential. Hyperexcitability and mutations of sodium channels are responsible for perception and transmission of inflammatory and neuropathic pain states. In this review, we describe the current knowledge of µ-CTX interacting with the different sodium channels subtypes, the mechanism of action and their potential therapeutic use as analgesic compounds in the clinical management of pain conditions.

## 1. Introduction

Cone snails are carnivorous and venomous molluscs belonging to the *Conus* genus (Figure S1) living mainly in the tropical marine areas. About 700 species of Cone snails express hundreds of peptide toxins collectively known as conotoxins (CTX) aimed to self-defense, competition and predation of other marine species by means of sting–structures that were reported to be fatal for human since from 300 years ago. CTX, however, do not exert only venomous activity but have a lot of pharmacological properties with specific bioactivity in the treatment of neurological disorders and the associated pain perception [1,2,3].

The presence of disulfide bonds is the essential characteristic for biological function of CTX that allow to divide CTX into two main categories, the disulfide-rich peptides and no-disulfide-rich ones; the first is mainly composed of a maximum of 30 amino acids and the second contains up to 80 amino acids. CTX are categorized into structural families based on the pattern of cysteine residues in terms of both number and position. Furthermore, differently from other peptides that may be subjected to poor absorption, proteolysis and biological half-lives, the presence of disulfide bonds confers to CTX a sort of stability based on the cross-linking between the cysteine side chains [4,5,6]. A further striking feature of CTX is the presence of a variety of posttranslational modifications which are, however, still to fully elucidate. CTX are used to act in a synergistic way to ensure that the venom exerts the most effective activity against the predated animals. The assemblage of CTX acting contemporarily has been named toxin cabal. Literature reports that different cabals co-exist, exerting different activities, including the modulation of different types of ion currents. 

Different distribution of ions across the plasma membrane gives rise to a trans-membrane potential known as resting potential (RP), which is negative in almost all cells studied. Ion currents are due to the flux of ions through ion channels, which are specific if it is allowed predominantly the passage of one ion species and may be gated in response to a change in voltage, defined voltage-operated channels. Ion currents are associated with a change in the RP that may shift towards more positive values, giving rise to the depolarisation of the plasma membrane [7].

**Figure 1:**
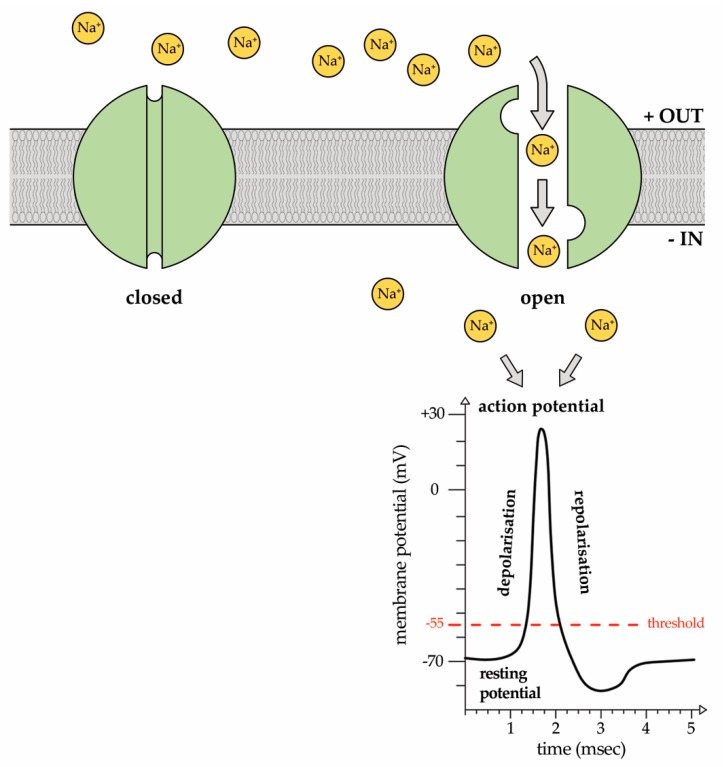
Representative image of the voltage-gated sodium channel (Nav) state. At the resting potential, the channel is closed. In response to a voltage change impulse greater than the threshold potential of −55 mV, the channel is activated and Na^+^ ions enter into the cytosol down their concentration gradient, giving rise to the action potential. It is a sudden, transient depolarization of the membrane potential that reaches a peak and, then, is followed by repolarization.

Voltage-gated sodium (Na^+^) channels (Nav channels) are responsible for the generation of the rapid depolarization of the membrane potential known as action potentials in excitable cells that, in turn, propagate electrical signals in muscles and nerves (Figure 1). 

Hence, Nav channel defects and mutations are associated with a wide range of neurological diseases known as channelopathies. Several CTX families have been identified to modulate Na^+^ current, in particular μ- and μO-CTX are antagonist of the Nav channels. This specificity has been used to discriminate different Nav channel subtypes, characterize specific binding sites on the channels and elucidate the μ-CTX-Nav channel complex interaction [8]. 

The aim of this review is to give an overview on the pharmacological activity that the µ-CTX superfamily exerts through the modulation of Na^+^ ion currents. A specific focus will be done on different physiological processes and mechanisms underlying neurological disorders and potential clinical application of these CTX in the therapeutic strategy for neuropathic pain alleviation. 

## 2. Sodium (Na^+^) Ion Currents

Discovery of Nav channels dates back to the 1950s [9] in the studies on the electric conductance in squid’s giant axon. Later on, Nav channels were isolated and purified in *Electrophorus electricus* electroplax membrane [10]. Recent advanced studies cloned different Nav channel subtypes. 

The role of Nav channels in the propagation of action potential in nerve, muscle and most of the excitable cells has stimulated intense research aimed to determine their structure and to clarify the basis of the voltage-dependent gating. The current recorded in the squid giant axon underlined by Nav channels lasted for a few milliseconds and was quickly inactivated, giving rise to a cascade of other ion currents activation aimed to restore the original potential. Following studies in the 1970s, a conceptual model of Na^+^ channel function was elaborated, defining also a detailed model of the selectivity of the Na^+^ channels (for review see [11]). Nav channel activators have been isolated from the venom of several animals, plants and bacteria, providing key insight into the pathophysiological roles of these channels [12].

Interestingly, these studies also established that drugs with anesthetic activity act on Na^+^ channels binding to a receptor located in the pore of the channel, through different mechanisms. Due to the crucial role of Nav currents in the transmission of electrical stimuli, their inhibitors have been largely used in clinical practice as anticonvulsant, antiarrhythmic and local anesthetic drugs. At present, the Nav channel family includes nine members encoded by Nav channel genes which share sequence homologies and that, due to their complex biochemistry, appear to be associated with many human diseases when down-regulated and/or mutated [13].

**Figure 2:**
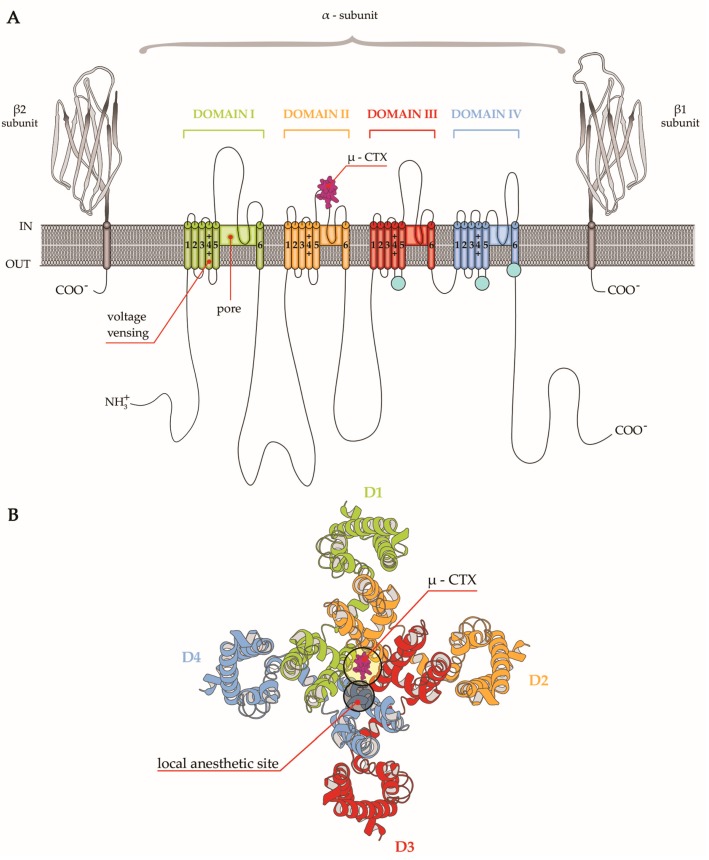
(**A**) Schematic representation of the sodium channel structure comprising a core α subunit and two auxiliary β subunits. The alpha subunit contains four homologue domains (Domain I-Domain IV), each consisting of six transmembrane helices (S1–S6) reported as cylinders. The pore of the channel is formed by S5 and S6 helices in DI, while the voltage sensor is formed by S1–S4 helices in DI. Auxiliary β subunits of the channels as immunoglobulin-like folds are illustrated. µ-CTX binding site is located between S5 and S6 helices in DII. (**B**) Schematic representation of the top view of the extracellular face of the α-subunit Nav channel. The location of the µ-CTX binding site and the close local anesthetic binding site are indicated.

Structurally, Nav channels are heteromeric complexes consisting of an α subunit of about 260s KDa coupled to one or two β subunits with lower weight. The subunits are single-chain peptides of about 2000 amino acids, which determines the differences between subtypes, and contain the receptors for toxins targeting the channel. In mammalian subtypes, the α subunits contain transmembrane and extracellular domains with high-sequence homology. Each domain is composed of six transmembrane helical segments named S1 to S6. The S4 segment present in every domain is the voltage sensor due to the richness in arginine and lysine and is responsible for the generation of the depolarization and the following return to the steady state. Segments 5 and 6 instead represent the Na^+^ pore and the filters to select Na^+^ passage. During a resting state, the channels are closed whereas, after depolarization of the RP, the segment S4 is alerted giving rise to a brief opening of the pore and Na^+^ passage (the open state) to quickly shift to an inactivated state. These main states are the basis for the sensitivity to drugs and inhibitors, which show different affinity for a specific state [14]. In the past, β subunits were considered as auxiliary of the α subunit; however, recent investigations have disclosed their multifunctional signaling role in physiological processes as cell adhesion, gene regulation and brain development [15] (Figure 2). 

Mutations in the genes encoding β subunits are linked to a number of diseases, including epilepsy, sudden death syndromes like sudden unexpected death in epilepsy, sudden infant death syndrome and cardiac arrhythmia. Although Nav channels β subunit-specific drugs have not yet been developed, this protein family is an emerging therapeutic target since it has been postulated that it may influence the kinetics of toxin block. From a pharmacological point of view, Na^+^ channel subtypes upon their diverse sensitivity to tetrodotoxin (TTX) can be distinguished as TTX-sensitive (the neuronal isoforms, Nav channels 1.1, 1.2, 1.3, 1.4, 1.6 and 1.7), or TTX-resistant (Nav channels 1.5, 1.8, 1.9) [16,17]. The role of Nav channels as analgesic targets has been deeply studied and highlighted with a specific focus on some specific isoforms. 

## 3. Na^+^ Currents—Linked Channelopathies

Channelopathies are diseases caused and underlined by disorders in ion channel functions whose etiology may be either genetic mainly due to ion mutations or acquired in cases of autoimmune insults, drugs and toxins [18]. Channelopathies can be found in many organ systems as cardiovascular, respiratory, endocrine, urinary, immune and nervous. In the latter, several neurological disorders such as epilepsy, cerebellar ataxia, myasthenia, myotonia, erythermalgia, schizophrenia, encephalopathy, Alzheimer syndrome, Dravet syndrome, and other neuropathies are associated with channels malfunctioning. Since ion currents are the flow of ions across the plasma membranes of either the cell or organelles, they play crucial roles in several cellular activities and in mechanisms of signal transduction in organs and related systems. Several channelopathies of the nervous system are underlined by Nav channel subtypes modulation. Literature reports that mutations of Nav channels 1.1 and 1.2 are linked to either epilepsy and the alteration of other central nervous system functions, whereas other Nav channel subtypes are mainly related to cardiac dysfunctions [19]. Neurological disorders, such as paralyses and cerebellar atrophy, are also associated with mutations in Nav channel subtypes (see for review [20]). In particular, nine isoforms according to the α-subunit sequence have been found in the central and peripheral nervous systems. The α-subtypes (Nav channels 1.1–1.9) present in sensory neurons underpin electrical activity through action potential propagation and this depolarization due to the influx of Na^+^ ions has been suggested to play a role in pain perception and transmission [21,22]. Although α subunits possess the features for Nav channel functioning, a co-expression of the β subunit was shown to influence channel gating, trafficking, expression and the biological activities of venom-derived toxins [23]. 

The anomalies in Na^+^ conductance due to injuries of different origin may lead to hyperexcitability of neurons resulting in neuropathic pain and disorders. In fact, channel defects and mutations have been related to vascular and painful organ diseases [24], whereas in other cases Nav channel mutations in functional sites are responsible for pain insensitivity [25]. At present, four channels seem to be strictly involved in pain disorders associated with several human pathologies from multiple sclerosis to cancer [26,27].

## 4. µ-CTX Modulating Nav Currents

**Figure 3:**
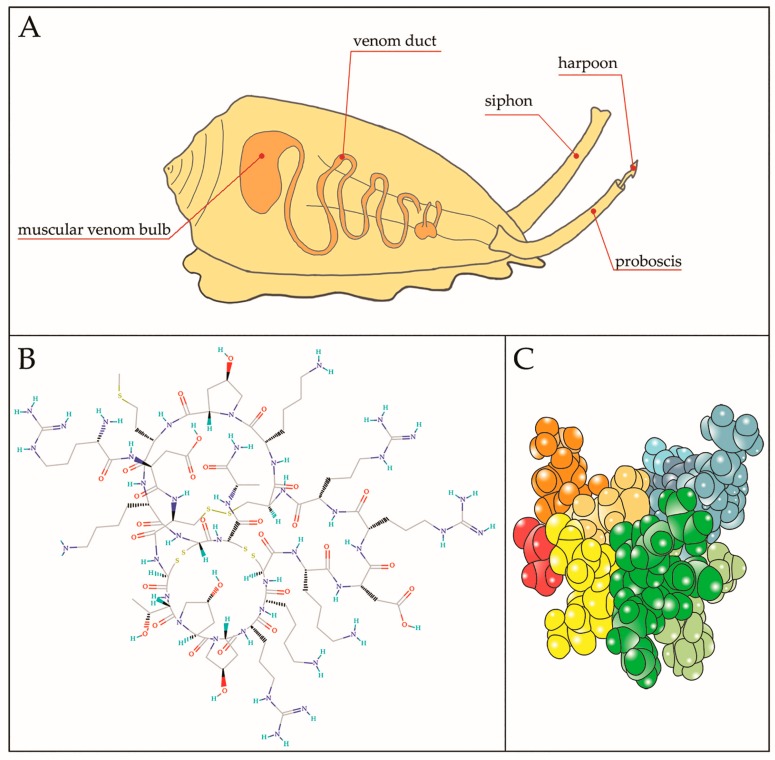
(**A**) Representative image of a cone snail predator showing internal venomous apparatus. The harpoon is the structure responsible for launching toxin and inducing paralysis of the prey. Chemical (**B**) and tridimensional (**C**) structure of µ-CTX-GIIIB, among the first toxin to be isolated from the venom of *Conus geographus*; B from https://pubchem.ncbi.nlm.nih.gov/compound/90469965#section=Top and (**C**) different colors indicate different residues.

The nine α subunits of Nav channels found in mammals are targets of toxins from marine animals. The most well-known inhibitors of Nav channels conductance are TTX and saxitoxin (STX), two non-peptidic neurotoxins isolated from puffer fish crustaceans, shellfish and other marine and terrestrial animals that exert different activity depending on the Nav channel subtype targeted [28,29]. These toxins exert high toxic effect and, furthermore, undergo a bioaccumulation in the tissues after ingestion of the animals as food. This concerning effect along with a resistance to the sodium channel proteins make these toxins not fully suitable for therapeutic use [30] although TTX is currently involved in Phase III trials for the treatment of cancer pain [14]. Due to the resistance of Nav channel subtypes to these toxins, intense investigations were aimed to identify new classes of toxins able to target these Nav channel subtypes [31]. Among the toxins that selectively link specific binding sites of Nav channels and share similar biological activities with both TTX and STX, there are three families of the neuroactive CTX: the μ, μO and δ, that induce respectively inhibition, blockage and delayed inactivation of the channels [32]. CTX exhibit a large amount of post-translational modifications, in particular related to the formation of disulfide bridges, which under the action of protein disulfide isomerases result in the formation of CTX isoforms [33]. The μ-conotoxins (μ-CTX) have been isolated from the venom of some species belonging to the genus Conus [34,35] and are characterized by the presence of paralytic peptides that affect mammalian neuromuscular transmission through a potent inhibition of α subunit of Nav channels. The occlusion of the ion-conducting pore of these channels occurs with a 1:1 stoichiometry in an all-or-none way and due to the presence of a guanidinium group as requisite for the pore-inhibition activity, μ-CTX together with the TTX and STX are classified as guanidinium toxins. Structurally, the μ-CTX is formed by 22 amino acids with six cysteines forming three inner disulfide bridges aimed to provide structural rigidity and stability of the global structure. The μ-CTX contain also a series of positively charged amino acids which are instrumental for their biological activity; in fact, if these residues are neutralized, the toxic activity results to be attenuated or is totally lost. First, μ-CTX were isolated from the venom of the *Conus geographus* and showed a preferential affinity for muscle subtype Nav channels (Figure 3). 

Later on, other μ-CTX with the affinity for neuronal subtypes Nav channels were isolated from other *Conus* species as *C. purpurascens, C. stercusmuscarum*, *C. striolatus, C. tulipa, C. kinoshitai, C. striatus*, *C. catus*, *C. magus* and *C. bullatus* [36,37,38]. 

In the last few years, studies on different CTX isoforms clarified either the molecular structures or their selectivity for the Nav channel subtypes. Specifically, μ- and μO-CTX constitute the family that selectively cause inhibition of Nav channels and that differ for the mechanism of inhibition of the current flowing across the channels. In particular, μ-CTX act through the direct block of the Nav channels pore whereas μO-CTX act by interfering with the voltage sensor [39]. Two main characterized isoforms of μ-CTX at physiological pH are the μ-GIIIA and μ-GIIIB from *Conus geographus*, which differ from each other at only four residues. The μ-GIIIA was the first μ-CTX characterized that targets mainly the skeletal muscle subtype Nav channel 1.4 [34,40,41,42]. Similar activity on Nav channel 1.4 subtype was exerted by μ-PIIIA that showed also an affinity with other ion channels as TTX-sensitive subtypes [43,44,45] and voltage-gated potassium channel subtypes of the KV1 family [46]. Recent findings also demonstrated that μ-PIIIA targets the bacterial voltage-gated sodium channel NaVAb, and uses multiple modes for binding and inhibiting it and Nav 1.4 with respect to the well-established pore blocking mechanisms. These authors constructed a profile showing that μ-PIIIA blocks NaVAb with subnanomolar affinity [43,47].

Later on, a group of μ-CTX targeting more selectively neuronal Nav channels were discovered and named μ-SmIIIA, μ-KIIIA and μ-SIIIA. It has been shown that they inhibit TTX-resistant Nav channels in vertebrates neurons [48] other than exerting similar action of μ-PIIIA on potassium channels [46] and, subsequently, to impact mammalian Nav channel subtypes [49]. An accurate structural and functional characterization of the μ-SIIIA, from *Conus striatus* demonstrated that this CTX is a potent, nearly irreversible neuronal blocker of Nav channels 1.2, and inhibitor of Nav channel 1.4 and Nav channel 1.6 at submicromolar concentrations with a potent analgesic action on mammalian neuronal Nav channel subtypes [50]. Although sharing several biochemical characteristics and sequence homology with μ-SIIIA, μ-SmIIIA from *Conus stercusmuscarum* appears to be a specific antagonist of TTX-resistant Nav channels exerting a potent and selective inhibition of Nav channels of adult rat small-diameter neurons [51,52]. The μ-KIIIA and μ-KIIIB from *Conus kinoshitai* are the shortest members of μ-CTX; however, they exert distinct activity by blocking neuronal Nav channels 1.1 and 1.2 [50]. The μ-CTX TIIIA was isolated from *Conus tulipa* and the sequence characterized was also confirmed by assay-guided fractionation of crude *Conus striatus* venom. The μ-TIIIA was shown to potently inhibit the dominant Nav channels 1.2 and Nav channels 1.4 isoforms present in the brain and not the TTX-sensitive channels expressed in dorsal root ganglia neurons [53]. 

Recent investigations led to the discovery of three μ-CTX, i.e., µ-BuIIIA, B and C from the fish-hunting species *Conus bullatus*. Although these exhibited different amino acid composition from known μ-CTX targeting the Nav channels 1.3 and 1.4, they were shown to potently inhibit the skeletal muscle isoforms [54,55]. Similarly, the three-disulfide-bridged CTX, μ-SxIIIA and µ-SxIIIB, isolated and characterized from the venom of *Conus striolatus*, were found to inhibit the skeletal muscle subtype Nav channels 1.2 and 1.4. However, μ-SxIIIA is also a potent blocker of the cloned mammalian Nav channel 1.4 expressed in Xenopus oocytes. 

The μ-CnIIIA, μ-CnIIIB, μ-CIIIC and μ-MIIIA, respectively from *Conus consor*, *catus* and *magnus,* share high degree of homology and block Nav channels 1 in amphibian neurons. However, they also exerted a various kind of selectivity for neuronal subtypes, especially when tested in mammalian systems [56,57,58]. 

**Figure 4:**
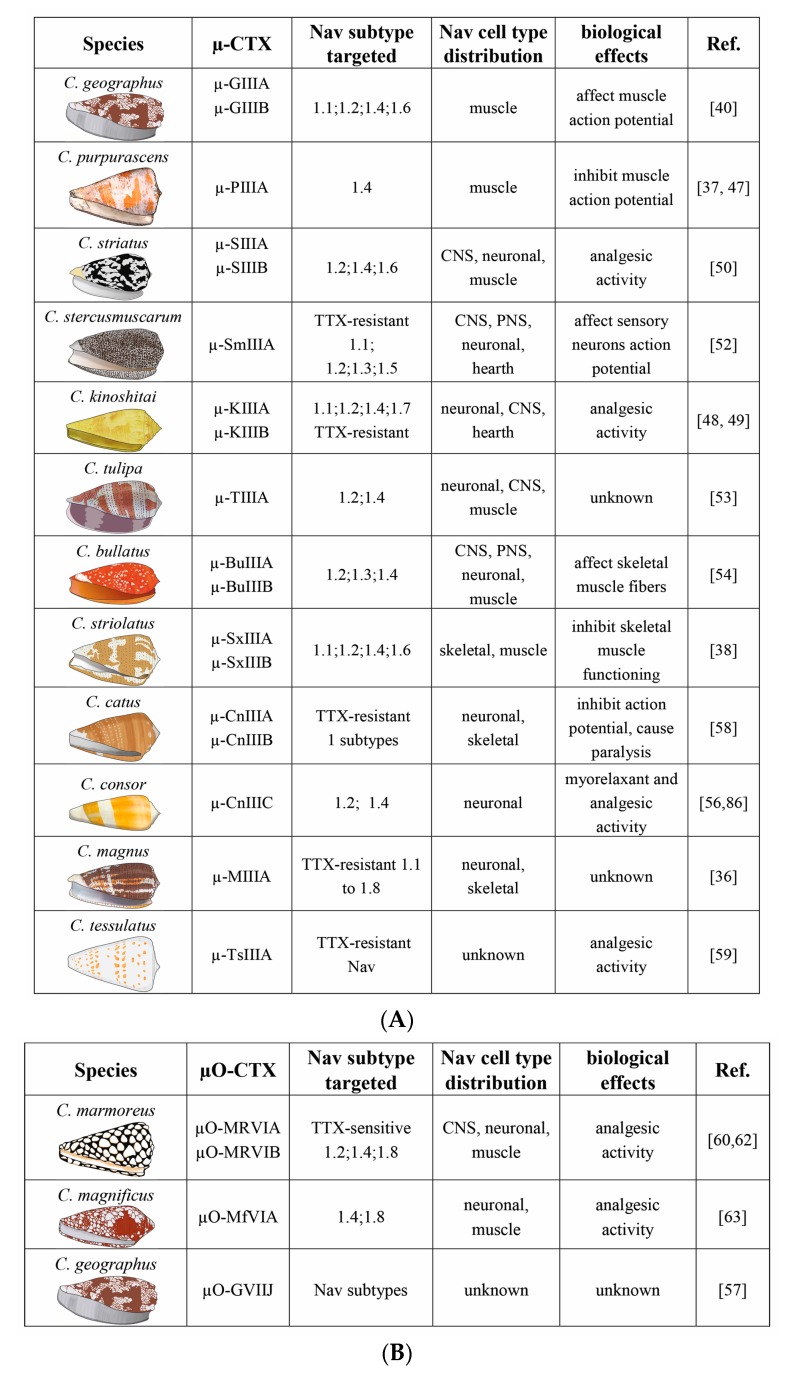
(**A**) The µ-CTX isolated from different species of the genus Conus, Nav channels targeted, their distributions in different tissues, and their biological effects, which are proved or extrapolated from channel activity data. CNS is central nervous system; PNS is peripheral nervous system. (**B**) The µO-CTX isolated from different species of the genus Conus, Nav channels targeted, their distributions in different tissues, and their biological effects, which are proved or extrapolated from channel activity data.

Very recent investigations identified a novel μ-CTX µ-TsIIIA from *Conus tessulatus*. By using patch clamp technique on rat neurons, it was shown that µ-TsIIIA inhibits TTX-resistant Nav channels but not TTX-sensitive Nav channels. Further investigations and mice hotplate analgesic assay indicated that µTsIIIA increased the pain threshold and exerted higher analgesic effects than other CTX, suggesting that that this toxin is a valuable compound for the development of new analgesic drugs [59] (Figure 4A,B).

The µO-CTX are an interesting class of CTX able to target either Nav channels or molluscan calcium channels. In particular, µO-MrVIA and µO-MrVIB are peptides from *Conus marmoreus*, composed of 31 residues and three disulfide bridges, and have been shown to be the first known peptidic inhibitors of the TTX-r Na^+^ current in rat neurons and of the TTX-sensitive Na^+^ currents. Since human TTX-resistant Nav channels are indicated as therapeutic targets for pain, the involvement of the µO-CTX is highlighted as potential leads for drug development [60]. These CTX are also known to selectively inhibit the TTX-insensitive Nav channel 1.8 isoform by exerting a relief persistent pain. In an attempt to elucidate the mechanism of action of these CTX, it was also shown an affinity for the Nav channel subtype 1.2 and 1.4 identifying C-terminal pore loop of domain-3 as the major determinant for subtype 1.4 being more inhibited than subtype 1.2. These results demonstrated that µO-CTX have a distinct molecular mechanism of channel inhibition with respect to µ-CTX [61]. Other authors also indicated that µO-CTX induced Nav channel inhibition acting on the voltage sensor [39,62].

Recently discovered and characterized was the µO-MfVIA, a novel μO-CTX from the venom of *Conus magnificus*. µO-MfVIA exhibited a high-sequence homology to previously known μO-CTX MrVIB. The biological activity of µO-MfVIA assessed by electrophysiological techniques and membrane potential-sensitive dyes showed a preferential inhibition of Nav channels 1.8 and 1.4 but also a lower affinity for other Nav channel subtypes [63]. Furthermore, a new μO-CTX GVIIJ from *Conus geographus* has been recently discovered. Its accurate characterization has shown a unique posttranslational modification and an odd number of cysteine residues in the primary amino acid sequence. Although the mechanism by which µO-GVIIJ may block the Nav channels is still to be clarified, it appears to be not a classical pore inhibitor [8,64].

## 5. µ-CTX Targeting Nav Channels in the Modulation of Pain States

Perception of pain helps animals and human to avoid injuries and physical damages. However, prolonged and intense painful sensation is a common debilitating condition source of intense suffering that may seriously interfere with daily life and normal functioning. Close to pain-associated pathologies, a genetic inherited pain syndrome has been evidenced by studying individuals and their familial pathophysiology. These studies showed that gene mutations of specific Na^+^ channels were responsible for most of the inherited pain sensitivity and insensitivity syndromes [65]. Noxious conditions are detected by nociceptors, sensory neurons that through propagation of action potentials allow the sensation of pain to reach the central nervous system [66].

Pain therapies are expensive for their clinical and socioeconomic impact due to medical treatments and the reduced productivity of workers affected by painful diseases [67]. Current medications with analgesic properties may have potential toxicities and limited efficacy and safety. These reasons reinforce the importance and the need to set up new pain treatments with low or null side effects. 

A variety of distinct origins and mechanisms underlie the pathophysiology of pain syndromes. Neuropathic pain may occur after nerve lesion or insult of the peripheral or central nervous system. These trigger molecular changes in neurons that become hypersensitive, developing an up-regulation of Na^+^ channels and receptors. Other pathological conditions as cardiac and muscle disorders up to recent investigation on cancer-associated pain have evidenced an unexpected role of Na^+^ channels in spontaneous and evoked types of pains [27,68] reinforcing the idea that isoform-specific modulators of these channels may provide novel approaches to treatment of pain [69].

Nav channel subtypes are differently distributed in the tissues and exhibit distinct biophysical and pharmacological properties. Being mediators of transmission of electrical signals, a change in their expression or activities generates neuropathic and inflammatory pain disorders. Studies from knockout mice and human mutations have indicated the strict involvement of four isoforms of Nav channels (1.3, 1.7, 1.8 and 1.9) in the heritable development and transmission of either acute or chronic pain [16]. Each subunit has a specialized property and function also underlined by different expression patterns. 

Nav channel 1.3 is expressed in the central nervous system with an expression level that is up-regulated in peripheral neurons in case of nerve fiber injury and inflammation, suggesting its involvement in pain sensation [70]. The down-regulation of Nav channel 1.3 expression in peripheral neurons resulted in a decreased hypersensitivity in neurons and pain perception. Among the painful diseases, involving Nav channel 1.3 trigeminal neuralgia has been identified. 

Nav channel 1.7 is the subunit predominant in the peripheral nervous system and the sensory neurons; therefore, it appears to be necessary for odor perception in rats, mice and humans. The induced mutations of the gene encoding this subtype give rise to a congenital insensitivity to pain, whereas gain-of-function mutagenesis experiments of this subtype generate distinct extreme pain disorders [71]. The discovery of human Nav channel 1.7 mutations that caused striking insensitivity to pain generated a renewed interest in the technologies aimed at drug discoveries and significant progress in the field [72]. The double action of Nav channel 1.7 in producing pain (primary erythromelalgia syndrome) and preventing pain (congenital analgesia) makes this subunit a potential therapeutic target and their inhibitors, interesting analgesic substances [73].

Nav channel 1.8 was shown to be expressed exclusively by primary afferent neurons [74] and functional characterization revealed that its expression occurred almost in all nociceptors [75]. Although a peculiar association with pathologies accompanied by persistent neuropathic pain states and inflammatory hyperalgesia were demonstrated [76], a precise role in pain transmission is not yet clear. Contrasting data are reported in literature on the role of Nav channel 1.8 in neuropathic pain. In fact, a reduced Nav channel 1.8 expression in damaged neurons suggests that this subunit is not involved in pain perception, whereas other authors showed that Nav channel 1.8 are redistributed to the axons of uninjured sciatic nerves after spinal nerve ligation, indicating a contribution to pain states. Furthermore, it was also shown that Nav channel 1.8 underlie nociception in the cold-related pain. From a molecular point of view, Nav channel 1.8 have been associated with altered β-subunit expression level.

The last subtype involved in chronic pain is Nav channel 1.9 expressed in the peripheral nervous system with a low-sequence homology to the other Nav channel subtypes. The mechanism of action demonstrated in Nav channel 1.9 null mice suggests its possible role in inflammatory pain; however, due to contrasting data, its specific action is still a matter of debate (see [77] for review). Mutations of gene encoding 1.8 and 1.9 Nav channel subunits may differently induce contrasting effect as small-fibre neuropathy and insensitivity to pain [78]. In this respect, µ-CTX being selective antagonists of Nav channels appear to be innovative and promising devices to promote pain relief [79]. A deep knowledge of Nav channel structure and binding sites has allowed to disclose the pharmacological potential of key compounds as toxins. Administration of compounds that reduce Nav channel activity have been used as antiepileptic, antiarrhythmic, and local anesthetic in clinical practice. Interestingly, it has been postulated that repeated stimulations of toxins may generate conformational changes in the receptors interfering with the gating of channels reducing their conductance and enhancing further interactions with the drug. The mechanism of gating modification instead of inhibition is at the basis of local anesthetics applications [80]. 

Validation of the role of Nav channels in pathophysiology of inherited or acquired pain states, soon clearly shows the potential therapeutic use of the µ-CTX targeting Nav channels in the treatment of chronic pain [81,82]. 

The μ-KIIIA was characterized as inhibitor of TTX-resistant Nav channels in amphibian neurons. However, following studies on mice demonstrated that μ-KIIIA blocked almost 80% of the TTX-sensitive, but only 20% of the TTX-resistant Nav channels. These studies based on the expression of Nav channels in Xenopus oocytes evidenced a potent analgesic activity in mouse pain model after systemic administration, showing for the first time that µ-CTX can block neuronal subtypes of mammalian Nav channels [49]. Similarly, µ-SmIIIA and µ-SIIIA showed a high degree of inhibition of TTX-sensitive Nav currents in mouse neurons. Further studies performing intraperitoneal administration of µ-SIIIA in a formalin-mediated inflammatory mouse pain model showed an analgesic effect even at low doses. However, different profiles of Nav channel inhibition indicated limits of the analgesic potential of µ-SIIIA [83]. Indirect evidences on the role of µCTX in the modulation of pain sensation come from a study aimed to identify Nav channel 1 isoforms responsible for action potentials in rat sciatic nerve [84,85].

The µ-CTX CnIIIC through the potent and selective antagonism of Nav channel 1.4 has been shown to elicit a block in rodents’ sciatic nerves and muscles emerging as a promising pharmacological tool in the development of myorelaxants and analgesics [56,86]. The recent findings of alternative modes by which µ-PIIIA binds Nav 1.4 channel also suggested a novel role of the binding properties for combating pain-associated diseases [43]. Due to the importance of understanding differences in the affinity and selectivity properties of CTX, recently, constructions of models of NaV1–µ-CTX complexes have been performed [87,88]. The µO-CTX MrVIB from *Conus marmoreus* was also displayed to have a substantial selectivity for Nav channels 1.8 and to exert the inhibition of pain behavior in rat models of persistent pain. These results indicated MrVIB as a promising lead compound for the treatment of both inflammatory and neuropathic chronic pain [62]. Similar analgesic activity has been proposed for the µO-CTX MfVIA from *Conus magnificus*. Due to the potent inhibition of Nav channels 1.4 and 1.8 abundant in dorsal root ganglion, it was proposed that µO-MfVIA may potently mediate pain relief [63].

## 6. Conclusions

A worldwide interest in the discovery of new analgesic compounds is due to the limited efficacy and unacceptable side effects of opioid-based pain therapies. These, in fact, causing constipation, emesis, dizziness, vomiting and seriously impacting driving and working activities, pose patients at risk of tolerance rather than mitigate their primary objective, that is, pain relief [89]. A major hurdle for this field is to identify excellent alternatives to opioids as analgesics in the costly pain therapy [90,91]. Modulators of Nav channel subtypes may represent new tools for facing pain signaling and disorders. The ample interest on Nav channels involvement for drug discovery and therapeutic treatment of pain [92,93] is supported by the findings that subunits 1.3, 1.7, 1.8 and 1.9 predominately expressed in sensory neurons are functionally involved in many different forms of pain. Thus, it is clear that µ-CTX, as inhibitors of Nav channels, are appropriate candidates to be administered to induce analgesia without undesirable side effects. 

This lesson comes from the unique CTX (ω-MVIIA) approved for clinical use and marketed for treatment of chronic pain (Prialt, the trade name) which acts by inhibiting calcium channels. Prialt exerts many side effects and, being administered by direct infusion in the spinal cord (intrathecally) is invasive; hence, it has been considered the last possibility for alleviation of chronic pain in clinical practice. Although µ-CTX targeting Nav channels have a systemic way of administration [82], there is still a paucity of high selective Nav channel blockers since the action on multiple subtypes may create side effects. 

This review is dealing with preclinical studies and there is a long way before a real therapeutic application. In fact, based on the advantages and the interest in CTX in pain therapies and the need for new drug design, further studies are required to investigate and demonstrate the pharmacological effectiveness of these compounds. However, new patents are currently reporting invention related to novel µ-CTX peptides, and/or biologically active fragments being possible candidates in pharmaceutical composition for the anesthetic medications [94].

In many cases, µ-CTX selectivity is still to be elucidated; hence, the hope is to discover new subtype-selective agents against Nav channels and create engineered analogues of therapeutic utility with decreased side effects, safety and the most noninvasive administration as the oral route [95].

New challenging perspective for structure-based drug discovery is at present to elucidate atomic structures of Nav channels in order to understand their function and mechanisms of action. Recent investigation by Huang [96] is, in fact, aimed to generate a homologous model of human Nav channel 1.7, to disclose disease-associated mutations. The search for new technical approaches are also in line with the fact that Conus species are threatened by increased pollution, climate change and overfishing. These conditions pose these mollusks at high risk of extinction in the years to come and their survival may be further compromised by the extraction of bioactive compounds described in this review. The important contribution of these animals in biomedicine and biotechnologies may, however, rely on new sustainable bio-molecular techniques as chemical synthesis and recombinant production in heterologous expression systems and polymerase chain reaction, sequencing of DNA fragments and transcriptomes that will allow in the future to obtain bioactive material with few or null animal sacrifice [97].

## Supplementary Materials

The following are available online at www.mdpi.com/1660-3397/15/10/295/s1. Figure S1: Different species of Conus genus.
