# Supplementary material for: µ-Conotoxins Modulating Sodium Currents in Pain Perception and Transmission: A Therapeutic Potential
Source: Mar Drugs. 2017 Sep 22;15(10):295. doi: 10.3390/md15100295 (PMC5666403; doi:10.3390/md15100295)
Supplement: Supplementary file 1 [file marinedrugs-15-00295-s001.pdf]

Supplementary Materials: μ-Conotoxins Modulating Sodium Currents in Pain Perception and Transmission: A Therapeutic Potential

Elisabetta Tosti, Raffaele Boni and Alessandra Gallo

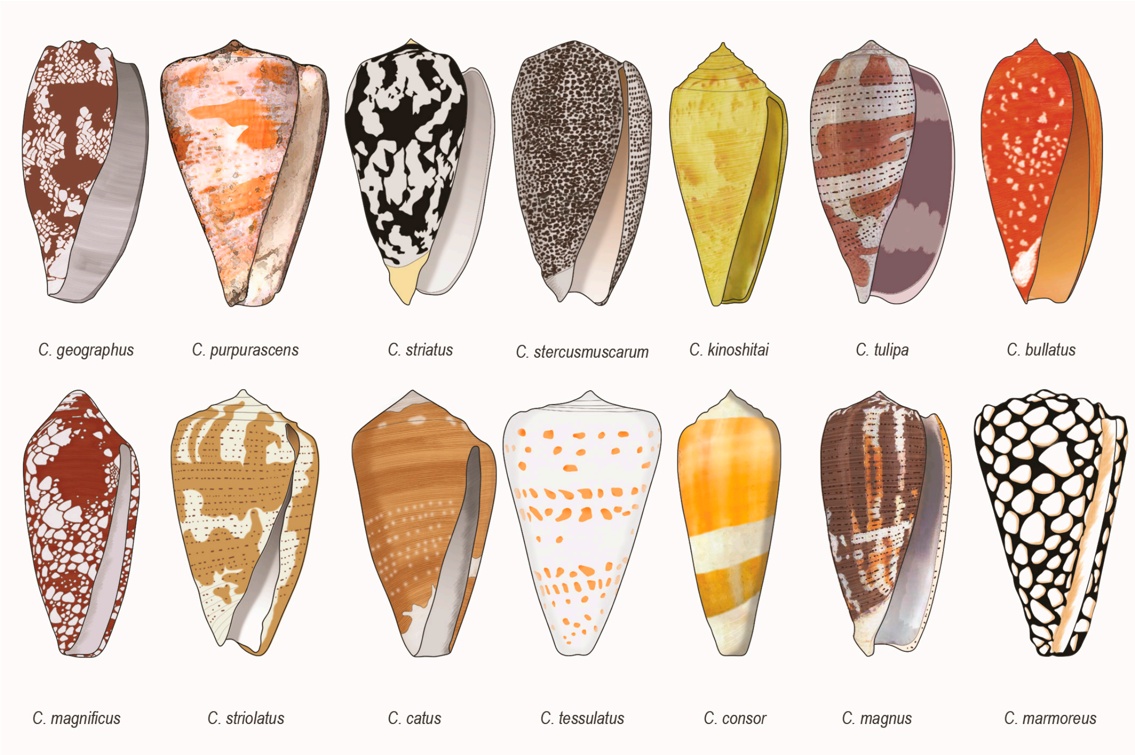

Figure S1. Different species of Conus genus.
